# Vehicle Dynamic Prediction Systems with On-Line Identification of Vehicle Parameters and Road Conditions

**DOI:** 10.3390/s121115778

**Published:** 2012-11-13

**Authors:** Ling-Yuan Hsu, Tsung-Lin Chen

**Affiliations:** Department of Mechanical Engineering, National Chiao Tung University, University Road 1001, Hsinchu, Taiwan; E-Mail: lance1214@gmail.com

**Keywords:** dynamics predictions, sensor fusion system, vehicle parameter identifications, road condition identifications

## Abstract

This paper presents a vehicle dynamics prediction system, which consists of a sensor fusion system and a vehicle parameter identification system. This sensor fusion system can obtain the six degree-of-freedom vehicle dynamics and two road angles without using a vehicle model. The vehicle parameter identification system uses the vehicle dynamics from the sensor fusion system to identify ten vehicle parameters in real time, including vehicle mass, moment of inertial, and road friction coefficients. With above two systems, the future vehicle dynamics is predicted by using a vehicle dynamics model, obtained from the parameter identification system, to propagate with time the current vehicle state values, obtained from the sensor fusion system. Comparing with most existing literatures in this field, the proposed approach improves the prediction accuracy both by incorporating more vehicle dynamics to the prediction system and by on-line identification to minimize the vehicle modeling errors. Simulation results show that the proposed method successfully predicts the vehicle dynamics in a left-hand turn event and a rollover event. The prediction inaccuracy is 0.51% in a left-hand turn event and 27.3% in a rollover event.

## 1. Introduction

In recent years, many vehicle control research propose using the future vehicle dynamics information to assist drivers' maneuvers. For example, the vehicle path predictions can provide the future position error for the vehicle guidance controls. Compared with the conventional “look-down” sensing system that provides current position error, the path prediction not only provides the information that are easier perceived by human drivers, but also provides additional information of road conditions, weather conditions, *etc.* [1,2]. As another example, many researchers propose using vehicle rollover predictions as anti-rollover measures [3–5]. The benefit is that the rollover prediction can trigger the control input earlier than the approaches without prediction. Consequently, the advance of the control action both saves actuation power and improves the driving safety. These examples highlight the importance of vehicle dynamics predictions.

In general, the future vehicle dynamics is predicted by using a vehicle mathematics model to numerically propagate current state values with time. Therefore, a vehicle dynamics prediction system needs a mathematic model and current vehicle state values [1–6]. There are two concerns regarding the mathematic model. First, many reports employ simplified vehicle models in the dynamics prediction, such as 2 DOF model [1], 4 DOF [2], and 2 DOF yaw-roll model [1–3,6]. The prediction results may only be acceptable for limited purposes and driving conditions. The inaccurate prediction result can be understood that, from the control viewpoint, the dynamics prediction is an open loop system. Thus, any model simplification would affect prediction accuracy to some extent. Our previous work [7] shows that even excluding the vehicle pitch dynamics from a 6 DOF vehicle model would result in an obvious error in the rollover prediction. One of the key components that are often neglected in the vehicle dynamics modeling is the road angle. Many research reports have shown that road angles have direct influences on the vehicle dynamics [8–10]. Our previous report [7] also show that, under the same driving maneuvers, a vehicle would roll over on a sloped road but would be under control on a flat road. Therefore, it is important to include the road angle in the vehicle dynamics prediction.

The second concern is the parameter uncertainty in the vehicle model. Most dynamics prediction methods use presumed vehicle parameters in the vehicle mathematics model [1–3,7]. This approach can simplify the prediction problem but may be inaccurate in practice. This is because the vehicle property (mass, moment of inertia) and road conditions (road angles, friction coefficients) may change from different driving situations, such as number of passengers, amount of fuel, road surface profiles, weather conditions, *etc.* Therefore, it is preferred that the parameters associated with the vehicle model can be identified in real time. From system identification viewpoint, the success of parameter identification depends on the model accuracy, persistent excitation, and signal-to-noise ratio (SNR) of the excitation signals [11]. Therefore, the vehicle parameter identification problem is closely related to the accompanied sensor fusion system that provides the measurements of the vehicle dynamics. In literatures, many vehicle parameter identification systems have been proposed to identify vehicle parameters such as vehicle masses, moments of inertia, road-tire frictions, *etc.* [9,12–14]. So far, we have not found a research report that identifies more than six vehicle parameters using their sensor systems.

As mentioned earlier, another key factor of the vehicle dynamics prediction is the sensor system for obtaining current vehicle state values. Since the vehicle system is highly nonlinear and many of its dynamics cannot be measured directly, the vehicle dynamics are often obtained by two ways: one is the observer-based sensor fusion system; the other is the kinematics-based sensor fusion system. The observer-based method needs a vehicle model and less number of sensors. On the contrary, the kinematics-based method does not require a vehicle model but needs more sensors. Since the state estimation accuracy of the observer-based method greatly relies on the incorporated model accuracy, the observer-based method is less preferred as compared with the kinematics-based method. Lots of kinematics-based sensor fusion systems employ a GPS and an IMU (three-axis accelerometer and three-axis gyroscope) to measure 6 degree-of-freedom (DOF) motions of an object. This sensor fusion system has been widely used in many applications, such as aircraft systems [15], ships [16], and road vehicles [17–19]. However, it has some drawbacks when applied to road vehicles. First, the rotation angles obtained by integrating the angular rates may suffer from the initial value ambiguity problem and the error accumulation problem. Second, both GPS and IMU are inertial sensors. Thus, the vehicle attitude and road angles are mingled together in the GPS and IMU measurements. Third, the measurement accuracy of the GPS is inadequate to determine the vehicle displacement in the vertical direction.

From pervious discussion, it can be concluded that a precise vehicle model is important for the accuracy of the dynamics prediction. How precise this vehicle model can be is determined by the accompanied sensor fusion system that provides current vehicle state values both for the state propagations and for the real time identification of vehicle parameters. In our previous work [20], we propose a kinematic based senor fusion system that employs a three-antenna global positioning system (GPS), an inertial measurement unit (IMU), and four suspension displacement sensors. This sensor fusion system can obtain the 6 DOF vehicle dynamics and two road angles. Based on this sensor system, we develop a vehicle dynamics prediction system in this paper using a vehicle model that is more complicated than most of the existing approaches. Moreover, the parameters of this vehicle model that may change in different driving conditions are identified in real time. In this paper, the dynamics prediction procedures, the parameter identification algorithms, the parameter observability, the SNR influence, and the prediction accuracy are all discussed in detail.

## 2. Euler Angles and Coordinate Systems

**Figure 1.**
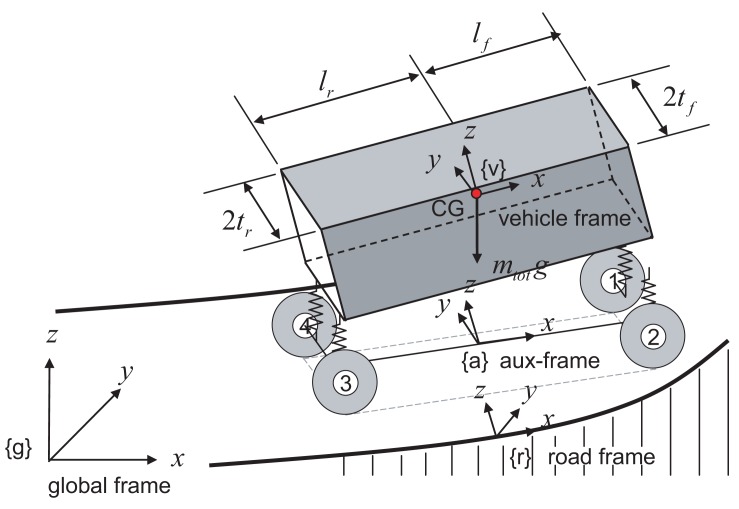
A schematic plot a vehicle and four coordinate systems (global frame, road frame, vehicle frame and auxiliary frame).

Three coordinate systems are introduced to describe a vehicle moving on a sloped road (see, Figure 1). These three coordinate systems are: global frame {g}, road frame {r}, and vehicle frame {v}. Similar to conventional research, the global frame is fixed to a point on Earth, while the vehicle frame is fixed to the center of gravity (CG) of the vehicle and rotates with the vehicle. The additional road frame is introduced to describe the vehicle dynamics on a sloped road, which is fixed to a road and rotated with the road terrain.

Three sets of Euler angles are used to describe the relationships between any two out of three coordinate systems. The first set of Euler angles (*ψ_g_, θ_g_, ϕ_g_*), which are referred to in this paper as the “absolute yaw angle”, “absolute pitch angle” and “absolute roll angle”, are used to describe the absolute attitude of the vehicle (global frame *vs.* vehicle frame). The rotation order of this set of Euler angles is yaw-pitch-roll. Its direction cosine matrix $\left({\text{C}}_{g}^{v}\right)$ can be written as follows:

(1) $$\begin{array}{ccc} {\text{C}}_{g}^{v} & = & \text{R}(x,\phi_{g})\;\text{R}(y,\theta_{g})\;\text{R}(z,\psi_{g})\\ \text{R}(x,\phi_{g}) & = & \left[\begin{array}{ccc} 1 & 0 & 0\\ 0 & \cos(\phi_{g}) & \sin(\phi_{g})\\ 0 & -\sin(\phi_{g}) & \cos(\phi_{g}) \end{array}\right]\\ \text{R}(y,\theta_{g}) & = & \left[\begin{array}{ccc} \cos(\theta_{g}) & 0 & \sin(\theta_{g})\\ 0 & 1 & 0\\ -\sin(\theta_{g}) & 0 & \cos(\theta_{g}) \end{array}\right]\\ \text{R}(z,\psi_{g}) & = & \left[\begin{array}{ccc} \cos(\psi_{g}) & \sin(\psi_{g}) & 0\\ -\sin(\psi_{g}) & \cos(\psi_{g}) & 0\\ 0 & 0 & 1 \end{array}\right] \end{array}$$

The second set of Euler angles (*θ_r_, ϕ_r_, ψ_r_*), which are referred to in this paper as the “road grade angle”, “road bank angle” and “road curve angle”, are used to describe the road grade profiles (global frame *vs.* road frame). The rotation order of this set of Euler angles is pitch-roll-yaw. Since a vehicle may move on a terrain irrelevant to the human-defined road path, it is impossible to determine the road curve angle from vehicle dynamics. Thus, it is assumed to be zero (*ψ_r_* = 0) for simplicity. Its direction cosine matrix $\left({\text{C}}_{g}^{r}\right)$ can be written as:

(2) $${\text{C}}_{g}^{r}=\text{R}(z,\psi_{r})\;\text{R}(x,\phi_{r})\;\text{R}(y,\theta_{r})$$

The third set of Euler angles (*ψ_v_, θ_v_, ϕ_v_*), which are referred to in this paper as the “vehicle yaw angle”, “vehicle pitch angle” and “vehicle roll angle”, are used to describe the vehicle attitude relative to a road plane (road frame *vs.* vehicle frame). The rotation order of this set of Euler angles is yaw-pitch-roll. Its direction cosine matrix $\left({\text{C}}_{r}^{v}\right)$ can be written as:

(3) $${\text{C}}_{r}^{v}=\text{R}(x,\phi_{v})\;\text{R}(y,\theta_{v})\;\text{R}(z,\psi_{v})$$

Since two sets of Euler angles are enough to describe the relationships between three coordinate systems, complying with the above angle definitions, the following relationship can be established for these angles:

(4) $${\text{C}}_{g}^{v}={\text{C}}_{r}^{v}\;{\text{C}}_{g}^{r}$$

An additional auxiliary frame (aux-frame) is obtained by rotating the z-axis of the road frame until the x-axis of the road frame is aligned with the x-axis of the body frame. The aux-frame is used because it describes vehicle translational motions in an intuitive manner while preserving the information of other vehicle dynamics relative to the road level. In the following vehicle modeling, vehicle translational motions are described in the aux-frame, and the rotational motions are described by angles *ψ_v_, θ_v_, ϕ_v_*.

## 3. A Sensor Fusion System for Road Vehicles

Since lots of the vehicle dynamics cannot be directly measured by individual sensors, a sensor fusion system is constructed to obtain the vehicle dynamics on a sloped road. The proposed sensor fusion system consists of a group of sensors, a kinematic model related to those sensor outputs, and a state estimation algorithm. They are discussed in the following.

### 3.1. Sensor Selections

#### 3.1.1. Three-Antenna GPS System

Different from a conventional GPS system, a three-antenna GPS is used here because it not only provides absolute position measurements $\left(x_{g}^{\text{gps}},y_{g}^{\text{gps}},z_{g}^{\text{gps}}\right)$but also absolute angle measurements $\left(\phi_{g}^{\text{gps}},\theta_{g}^{\text{gps}},\psi_{g}^{\text{gps}}\right)$. Both information are relative to the global frame, and the reported angle measuring error is approximately 0.1° from a test vehicle [21,22].

#### 3.1.2. Suspension Displacement Sensors

Four suspension displacement sensors are installed at four corners of a vehicle. The suspension deflection can be related to the vehicle attitude and vertical displacement of the vehicle CG, both relative to the road frame:

(5) $$\begin{array}{ccc} z_{r}^{\text{sus}} & = & \frac{-l_{r}\left(H_{1}^{\text{sus}}+H_{2}^{\text{sus}}\right)-l_{f}\left(H_{3}^{\text{sus}}+H_{4}^{\text{sus}}\right)}{2l_{f}+2l_{r}}\\ \theta_{v}^{\text{sus}} & = & \sin^{-1}\left\{\frac{H_{1}^{\text{sus}}+H_{2}^{\text{sus}}-H_{3}^{\text{sus}}-H_{4}^{\text{sus}}}{2l_{f}+2l_{r}}\right\}\\ \phi_{v}^{\text{sus}} & = & \sin^{-1}\left\{\frac{-H_{1}^{\text{sus}}+H_{2}^{\text{sus}}+H_{3}^{\text{sus}}-H_{4}^{\text{sus}}}{\left(2t_{f}+2t_{r}\right)\cos\theta_{v}}\right\} \end{array}$$

where the superscript (*sus*) denotes the physical quantities measured by the suspension displacement sensors; *z_r_* is the vertical displacement of the vehicle CG observed in the road frame {r}; *H_i_* represents the displacement of suspension at the corner *i*; the subscript (*i*) refers to four suspension corners in a way: 1 → front-left, 2 to 4 in a clockwise direction; l_f_ and l_r_ are the distances from CG to the front and rear axis, respectively; t_f_ and t_r_ are one half of the distances of the front and rear track, respectively.

#### 3.1.3. Inertial Measurement Unit

An IMU sensor is installed at the center of gravity of the vehicle to measure the 6 DOF movements. They are used here to improve the estimation accuracy of the vehicle dynamics.

### 3.2. A Kinematic Model

As discussed in this paper, three sets of Euler angles (nine angles in total) parameterize this vehicle attitude determination system. The relationships stated in Equation (4) provide three constrained equations; the 3-antenna GPS system provides the measurements of three absolute vehicle angles (*ϕ_g_, θ_g_, ψ_g_*); the measurements from suspension displacement sensors provide the values of two vehicle attitude $\left(\phi_{v}^{\text{sus}},\theta_{v}^{\text{sus}}\right)$; the road curve angle is assumed to be zero (*ψ_r_* = 0). Therefore, even without a kinematic model, those nine angles can be solved.

In addition to the angle measurements above, the vehicle rotational dynamics are also present in the IMU measurements, GPS position measurements, and suspension displacement measurements. In order to improve the robustness and accuracy of the angle determination, all the sensor measurements should be used. Thus, the estimation of vehicle dynamics is done for the rotational dynamics and translational dynamics simultaneously. In that case, since Equation (4) provide three constrained equations, six angles must be employed as system states to describe the vehicle attitude, and those six angle states are chosen as (*ϕ_g_, θ_g_, ψ_g_, ϕ_r_, θ9_r_, ψ_v_*) for the ease of subsequent fusion algorithm derivation. Furthermore, in order to apply existing state estimation techniques to this problem, the “governing equations” of these unknown angles must be obtained beforehand and added to the conventional kinematic model. Since it is impractical to either use additional sensors to measure the change rate of the last three angles or obtain this information for a specific case, the change rates of the last three angles are assumed to be zero.

(6) $$\begin{array}{c} {\dot{\phi }}_{r}=0\\ {\dot{\theta }}_{r}=0\\ {\dot{\psi }}_{v}=0 \end{array}$$

Thus, a kinematic model that can coordinate the outputs of IMU, GPS and suspension displacement sensors is:

(7) $$\begin{array}{cc} \dot{x} & =f(x,u)\\ & =Fx+G_{\text{acc}}\;\left[\begin{array}{c} A_{x}^{\text{acc}}\\ A_{y}^{\text{acc}}\\ A_{z}^{\text{acc}} \end{array}\right]+G_{\text{gyro}}\;\left[\begin{array}{c} \omega_{x}^{\text{gyro}}\\ \omega_{y}^{\text{gyro}}\\ \omega_{z}^{\text{gyro}} \end{array}\right]\\ x & ={\left[x_{g},y_{g,}z_{g},{\dot{x}}_{g},{\dot{y}}_{g,}{\dot{z}}_{g},\phi_{g},\theta_{g},\psi_{g},\ldots \phi_{r},\theta_{r},\psi_{v}\right]}^{T}\\ u & ={\left[A_{x}^{\text{acc}},A_{y}^{\text{acc}},A_{z}^{\text{acc}},\omega_{x}^{\text{gyro}},\omega_{y}^{\text{gyro}},\omega_{z}^{\text{gyro}}\right]}^{T}\\ & F=\left[\begin{array}{ccc} 0_{3\times 3} & I_{3\times 3} & 0_{3\times 6}\\ & 0_{9\times 12} & \end{array}\right]\\ & G_{\text{acc}}={\left[0_{3\times 3}{C_{g}^{v}}^{-1}0_{6\times 3}\right]}^{T}\\ & G_{\text{gyro}}={\left[0_{3\times 6}\;{C_{\omega }}^{-1}\;0_{3\times 3}\right]}^{T}\\ & C_{\omega }=\left[\begin{array}{ccc} 1 & 0 & -\sin\theta_{g}\\ 0 & \cos\phi_{g} & \cos\theta_{g}\sin\phi_{g}\\ 0 & -\sin\phi_{g} & \cos\theta_{g}\cos\phi_{g} \end{array}\right] \end{array}$$

where $\left(A_{x}^{\text{acc}},A_{y}^{\text{acc}},A_{z}^{\text{acc}}\right)$ represents the measurements from a three-axis accelerometer; $\left(\omega_{x}^{\text{gyro}},\omega_{y}^{\text{gyro}},\omega_{z}^{\text{gyro}}\right)$ represents the measurements from a three-axis gyroscope; (*x_g_, y_g_, z_g_*) and (*ẋ_g_, ẏ_g_, ż_g_*) represent position and velocity observed in the global frame; C*_ω_* describes the relation between angular rate and rate of change of Euler angles, which can be found in [23].

### 3.3. State Estimation Algorithm

For a dynamics model shown in Equation (7), a state observer that can estimate each state value is constructed as follows:

(8) $$\dot{\hat{x}}=f(\hat{x},u)+\text{L}(h(x)-h(\hat{x}))$$

where the (ˆ) denotes the estimated state value; h(x) is the system output equation; **L** is the matrix of observer gains. In this paper, the extended Kalman filter is chosen as the state estimation algorithm to calculate the observer gain. The standard procedures of the extended Kalman filter can be found in [11].

The system output equation h(x) in Equation (8) is carefully chosen as follows to ensure the system observability.

(9) $$\begin{array}{cc} h(x) & ={\left[y_{1}^{\text{gps}},y_{2}^{\text{gps}},y_{1}^{\text{sus}},y_{2}^{\text{sus}}\right]}^{T}\\ y_{1}^{\text{gps}} & =\left[x_{g}^{\text{gps}},y_{g}^{\text{gps}},z_{g}^{\text{gps}},\phi_{g}^{\text{gps}},\theta_{g}^{\text{gps}},\psi_{g}^{\text{gps}}\right]\\ y_{2}^{\text{gps}} & =\left[C_{g}^{v}(1,1),C_{g}^{v}(1,2)\right]\\ & =\left[C_{r}^{v}C_{g}^{r}(1,1),C_{r}^{v}C_{g}^{r}(1,2)\right]\\ y_{1}^{\text{sus}} & =\left[C_{r}^{v}(1,3),C_{r}^{v}(2,3),C_{r}^{v}(3,3)\right]\\ & =\left[{C_{g}^{v}C_{g}^{r}}^{-1}(1,3),{C_{g}^{v}C_{g}^{r}}^{-1}(2,3),{C_{g}^{v}C_{g}^{r}}^{-1}(3,3)\right]\\ y_{2}^{\text{sus}} & =z_{r}^{\text{sus}}\\ & =C_{g}^{r}(3,1)x_{g}+C_{g}^{r}(3,2)y_{g}+C_{g}^{r}(3,3)z_{g} \end{array}$$

where C(*m, n*) denotes the element in the *m*th row and the *n*th column of the matrix C. The output equation $y_{1}^{\text{gps}}$ provides the locations and attitude of the vehicle in the global frame and its values is obtained from the measurements of a three-antenna GPS. The output equation $y_{2}^{\text{gps}}$ is a function of (*ϕ_r_, θ_r_, ψ_v_*) and its values are calculated from the measurements of a three-antenna GPS. The output equation $y_{1}^{\text{sus}}$ is a function of (*ϕ_r_, θ_r_*) and its values are calculated from the measurements of the suspension displacement sensors. The output equation $y_{2}^{\text{sus}}$ is related to a function of (*ϕ_r_, θ_r_*) and (*x_g_, y_g_, z_g_*) and its values are then calculated from the measurements of the suspension displacement sensors in Equation (5) and three-antenna GPS.

It should be emphasized that the $\left(y_{2}^{\text{gps}},y_{1}^{\text{sus}}\right)$ are two sets of output equations, and each consists of 2 to 3 nonlinear algebraic equations. Each equation consists of multiplication terms of two or more trigonometric functions from the corresponding direction cosine matrix. In most cases, only one equation in each set of output equations is enough to ensure the observability of state estimations. However, since it involves multiplications of trigonometric functions, the estimation would fail at certain angles. Therefore, redundant equations are used to ensure the success at all angles.

### 3.4. Multi-Rate Kalman Filter

Since the outputs of the GPS, IMU and suspension displacement sensors are unsynchronized and contaminated by different noise characteristics (see Table 1), instead of using a conventional extended Kalman filter, a multi-rate extended Kalman filter [24] is chosen to coordinate these sensor outputs. The algorithm of a multi-rate Kalman filter is similar to that of a conventional extended Kalman filter with the only difference in correcting the estimated state values. When the GPS measurement is available, the estimated state value is updated by the measurements of the GPS and the suspension displacement sensors. When the GPS measurement is unavailable, the estimated state value is updated only by the measurements of the suspension displacement sensors. It is done by the following:

**when GPS measurements are available,**

(10) $$\begin{array}{c} h(x)={\left[y_{1}^{\text{gps}},y_{2}^{\text{gps}},y_{1}^{\text{sus}},y_{2}^{\text{sus}}\right]}^{T}\\ h(\hat{x})={\left[{\hat{y}}_{1}^{\text{gps}},{\hat{y}}_{2}^{\text{gps}},{\hat{y}}_{1}^{\text{sus}},{\hat{y}}_{2}^{\text{sus}}\right]}^{T} \end{array}$$

**when GPS measurements are unavailable,**

(11) $$\begin{array}{c} h(x)={\left[0_{1\times 6},0_{1\times 2},y_{1}^{\text{sus}},y_{2}^{\text{sus}}\right]}^{T}\\ h(\hat{x})={\left[0_{1\times 6},0_{1\times 2},{\hat{y}}_{1}^{\text{sus}},{\hat{y}}_{2}^{\text{sus}}\right]}^{T} \end{array}$$

### 3.5. Alpha-Beta Filter

The above state estimation process can provide noiseless information for the displacement, velocity, and attitude of the vehicle, but not for the angular velocity and accelerations. Without this information, the subsequent vehicle parameter identification can be much complicated. Potentially, the angular velocity and accelerations can also be obtained from Kalman filtering by including those two states as system states in Equation (7). However, this approach may need a fictitious noise and increase the computation complexity. Hence, the alpha-beta filter is used to obtain the values for the angular velocity and acceleration.

The alpha-beta filter is a steady-state filter for noisy signals. Its algorithm is shown as follows:

(12) $$\begin{array}{ll} {\hat{x}}_{\alpha }(k+1) & ={\text{A}}_{\alpha }{\hat{x}}_{\alpha }(k)+{\text{K}}_{\alpha }(k+1)[z(k+1)-\hat{z}(k+1)]\\ {\text{A}}_{\alpha } & =\left[\begin{array}{cc} 1 & T_{s}\\ 0 & 1 \end{array}\right]\\ {\text{K}}_{\alpha }(k+1) & ={\left[\alpha ,\beta /T_{s}\right]}^{T} \end{array}$$

where x̂*_α_* is a set of state vector for a two-dimensional model; *z* is the measured first dimensional state; *z* is the estimated value of *z*; **A***_α_* is the system matrix of the two-dimensional model; *T_s_* is the sampling period; the feedback gains *α* and *β* are chosen empirically. The detailed information of alpha-beta filters can be found in [11]. For example, x̂*_α_* can be chosen as [*ω_x_, ω̇_x_*]^T^; the corresponding *z* is the measured rotational velocity from the gyroscope $\left(\omega_{x}^{\text{gyro}}\right)$. Thus, through the alpha-beta filter, the rotational acceleration can be obtained without the direct differentiation.

## 4. A Vehicle Model for the Dynamics Predictions

As mentioned earlier, a dynamics prediction system needs a precise vehicle model. Furthermore, some of the parameters in that vehicle model should be identified in real time. To meet both requirements, we propose the following vehicle model for the dynamics prediction, which consists of 6 DOF vehicle dynamics, road angles, tire-road friction, nonlinear suspension, *etc.*

(13) $$\begin{array}{cc} m_{\text{tot}}\left({\ddot{x}}_{a}-{\dot{y}}_{a}{\dot{\psi }}_{v}\right) & =F_{x,\text{tire}}+m_{\text{tot}}G_{x}\\ m_{\text{tot}}\left({\ddot{y}}_{a}+{\dot{x}}_{a}{\dot{\psi }}_{v}\right) & =F_{y,\text{tire}}+m_{\text{tot}}G_{y}\\ m_{\text{tot}}{\ddot{z}}_{a} & =F_{z,\text{spring}}+m_{\text{tot}}G_{z}\\ & I_{x}{\dot{\omega }}_{x}=\left(I_{y}-I_{z}\right)\omega_{y}\omega_{z}+M_{x}\\ & I_{y}{\dot{\omega }}_{y}=\left(I_{z}-I_{x}\right)\omega_{z}\omega_{x}+M_{y}\\ & I_{z}{\dot{\omega }}_{z}=\left(I_{x}-I_{y}\right)\omega_{x}\omega_{y}+M_{z}\\ & I_{\omega }{\dot{\omega }}_{i}=-r_{i}F_{a,\text{tire},i}+T_{i}\left(i=1∼4\right)\\ & \left[\begin{array}{c} G_{x}\\ G_{y}\\ G_{z} \end{array}\right]=R\left(z,\psi_{v}\right)R\left(x,\phi_{r}\right)R\left(y,\theta_{r}\right)\left[\begin{array}{c} 0\\ 0\\ -g \end{array}\right] \end{array}$$

where *g* is the Earth gravity; (*x_a_, y_a_, z_a_*) represents the three-axis displacement of the vehicle CG observed in the aux-frame; (*F*_x,tire_, *F*_y,tire_, *F*_z,spring_) are the translational forces generated by tires and suspension systems; (*M_x_, M_y_, M_z_*) are the external torques acting on the vehicle CG along three axes of the vehicle frame, which are the functions of forces (*F*_x,tire_, *F*_y,tire_, *F*_z,spring_), vehicle attitude (*ψ_v_, θ_v_, ϕ_v_*), and vehicle geometry [25]; (*I_x_, I_y_, I_z_*) are the moment of inertia of the vehicle body along three axes of the vehicle frame; (*ω_x_, ω_y_, ω_z_*) are the rotational velocities of the vehicle body along three axes of the vehicle frame; *ω_i_* represents the angular rate of each tire *i; F*_a,tire_,*_i_* is the longitudinal adhesive force generated by tire *i; T_i_* are the wheel torque transmitted to the tire *i; r_i_* is the effective rolling radius of a tire *i; I_ω_* is the moment of inertia of a tire.

The suspension system is modeled as a nonlinear spring-mass-damper system. Thus, the translational force generated by the suspension can be described as follows [7]:

(14) $$\begin{array}{cc} F_{z,\text{spring},i} & =K_{s,i}H_{i}+D_{s,i}{\dot{H}}_{i}+m_{u,i}g\\ K_{s,i} & =\begin{array}{cc} C_{1}e^{C_{2}\left(H_{i}-C_{3}\right)} & \left(i=1∼4\right) \end{array}\\ H_{i} & =\{\begin{array}{ccc} H_{i}, & \text{for} & H_{i}>-m_{u,i}g/K_{s,i}\\ -m_{u,i}g/K_{s,i}, & \text{for} & H_{i}\leq -m_{u,i}g/K_{s,i} \end{array} \end{array}$$

where *K_s,i_* is the spring stiffness of the suspension *i* and *C*_1_, *C*_2_, *C*_3_ parameterize the stiffness; *D_s,i_* is the damper coefficient of the suspension *i; m_u,i_* is the unsprung mass of the suspension corner *i*.

The adhesive force generated by tire is a highly nonlinear function of variables including slip ratios, slip angles, vertical loads, *etc.* [26,27]. However, under normal vehicle maneuvering, the adhesive force is almost linearly proportional to those variables. Thus, a linear tire model [13,14] is used to describe the longitudinal and lateral tire forces (*F*_a,tire_, *F*_b,tire_) for simplicity.

(15) $$\begin{array}{c} F_{a,\text{tire},i}=C_{\lambda ,i}\lambda_{i}\\ F_{b,\text{tire},i}=C_{\alpha ,i}\alpha_{i} \end{array}$$

where *C_λ,i_* and *C_α,i_* are the tracking stiffness and the cornering stiffness of the tire *i*, respectively; *λ_i_* and *α_i_* are the slip ratio and slip angle, respectively. The translational forces represented in the x-axis and the y-axis of the aux-frame (*F*_x,tire_, *F*_y,tire_) can be obtained as follows:

(16) $$\begin{array}{c} F_{x,\text{tire}}=\sum \left(F_{a,\text{tire},i}\cos\delta_{i}-F_{b,\text{tire},i}\sin\delta_{i}\right)\\ F_{y,\text{tire}}=\sum \left(F_{a,\text{tire},i}\sin\delta_{i}+F_{b,\text{tire},i}\cos\delta_{i}\right). \end{array}$$

Noted that two rear wheel angles (*δ*_3_, *δ*_4_) are zeros for a front-steer vehicle, and two front wheel angles (*δ*_1_, *δ*_2_) are known values because they can be obtained by the steering wheel angle and the Ackerman principle [28]. The vehicle model for the dynamics prediction is thus constructed and shown in Equations (13)–(16).

## 5. Vehicle Parameter Identification Systems

In this approach, the parameters shown in the vehicle model in Equations (13)–(16) needs to be identified using the vehicle dynamics obtained from the sensor fusion system in Equation (7). Note that the vehicle dynamics from the sensor fusion system is presented in the global frame, while the above vehicle model is presented in the aux-frame. Thus, the estimated vehicle dynamics are transformed into the aux-frame using the matrices shown in Equations (1)–(3), prior to the vehicle parameter identification.

After feeding the vehicle dynamics, Equation (13) becomes a set of 10 linear equations with 12 unknown vehicle parameters. Hence, the number of the cornering stiffness is reduced from four to two because the cornering stiffness is similar at two sides of the vehicle [13,14]. In that case, the model of the lateral tire force is simplified as follows:

(17) $$\begin{array}{cc} F_{b,\text{tire},f} & =F_{b,\text{tire},1}+F_{b,\text{tire},2}\\ & ≃C_{\alpha ,f}\left(\alpha_{1}+\alpha_{2}\right)/2\\ F_{b,\text{tire},r} & =F_{b,\text{tire},1}+F_{b,\text{tire},2}\\ & ≃C_{\alpha ,r}\left(\alpha_{3}+\alpha_{4}\right)/2 \end{array}$$

where

$$\begin{array}{c} C_{\alpha ,f}=C_{\alpha ,1}+C_{\alpha ,2}\\ C_{\alpha ,r}=C_{\alpha ,3}+C_{\alpha ,4}. \end{array}$$

### 5.1. Recursive Least-Square Algorithms

In order to apply the recursive-least-square (RLS) algorithm to identify vehicle parameters, the vehicle parameters and the corresponding measured dynamics are rearranged into the following format:

(18) $${\text{QA}}_{\text{rls}}{\text{WW}}^{-1}\bar{x}=\text{Qb}$$

where, x̄ represent the vehicle parameters; **A***_rls_* and **b** are the vehicle dynamics both from direct sensor measurements and the output of the sensor fusion system; **Q** is the weighting matrix; **W** is the scaling matrix. By choosing matrices **Q**, **W**, and an initial guess of the vector x̄, the vector x̄ is solved recursively by the following steps [29]:

(19) $$\begin{array}{lll} \bar{\text{P}}(k+1) & =\bar{\text{P}}(k)– & \bar{\text{P}}(k){\text{A}}_{Q}{\left(k+1\right)}^{\text{T}}{\left[\text{I}+{\text{A}}_{Q}(k+1)\cdot \bar{\text{P}}(k){\text{A}}_{Q}{\left(k+1\right)}^{T}\right]}^{-1}{\text{A}}_{Q}(k+1)\bar{\text{P}}(k)\\ {\bar{x}}_{Q}(k+1) & = & {\bar{x}}_{Q}(k)-\bar{\text{P}}(k+1){\text{A}}_{Q}{\left(k+1\right)}^{\text{T}}\cdot \left[Q(k+1)b(k+1)-{\text{A}}_{Q}(k+1){\bar{x}}_{Q}(k)\right]\\ {\text{A}}_{Q}(k+1) & = & Q(k+1){\text{A}}_{\text{rls}}(k+1)\text{W}(k+1)\\ {\bar{x}}_{Q}(k+1) & = & \text{W}{\left(k+1\right)}^{-1}\bar{x}(k+1) \end{array}$$

where **P̄** is the covariance matrix.

### 5.2. Vehicle Parameter Identifications

The following vehicle parameters are identified using the above RLS algorithms: vehicle mass (*m_tot_*), moment of inertia of the vehicle body (*I_x_, I_y_, I_z_*), tracking stiffness (*C*_λ,1∼4_), and the cornering stiffness (*C_α,f_, C_α,r_*). These parameters are identified in real time because their values can be changing in each driving condition. The other vehicle parameters such as the spring stiffness *K_s_*, the damper coefficient *D_s_*, the unsprung mass *m_u_*, the rolling radius of tire *r*, and the moment of inertia of the tire *I_ω_* are assumed to be known values.

In this case, it is possible to manipulate the signal processing steps and formulate four independent RLS problems for identifying the above ten parameters, which can greatly reduce the computation loads and efforts of searching the optimal **Q** and **W** matrices. Those four independent RLS problems are “mass identification”, “tracking stiffness identification”, “cornering stiffness identification”, and “moment of inertia identification”.

#### 5.2.1. Mass Identification

The translational dynamics in z direction in the vehicle model in Equations (13) and (14) can be rearranged as:

(20) $$q_{11}A_{\text{rls},1}w_{11}^{-1}{\bar{x}}_{1}=q_{11}b_{1}$$

where

$$\begin{array}{lll} {\bar{x}}_{1} & ≜ & {\bar{m}}_{\text{tot}}\\ A_{\text{rls},1} & ≜ & {\ddot{\hat{z}}}_{a}-{\hat{G}}_{z}\\ b_{1} & ≜ & \sum_{i=1}^{4}K_{s}H_{i}^{\text{sus}}+D_{s}{\dot{H}}_{i}^{\text{sus}}+m_{u,i}g. \end{array}$$

where *q*_11_ and *w*_11_ are the elements in weighting and scaling matrices, respectively. ${\dot{H}}_{i}^{\text{sus}}$ is the derivative of the suspension displacement and is obtained by the alpha-beta filter via the input from the suspension displacement measurements.

#### 5.2.2. Tracking Stiffness Identification

The wheel dynamics in the vehicle model in Equations (13) and (15)–(16) can be rearranged as:

(21) $${\text{Q}}_{2}{\text{A}}_{\text{rls},2}{\text{W}}_{2}{\text{W}}_{2}^{-1}{\bar{x}}_{2}={\text{Q}}_{2}b_{2}$$

where

$$\begin{array}{lll} {\bar{x}}_{2} & ≜ & {\left[{\bar{C}}_{\lambda ,1},{\bar{C}}_{\lambda ,2},{\bar{C}}_{\lambda ,3},{\bar{C}}_{\lambda ,4}\right]}^{T}\\ {\text{A}}_{\text{rls},2} & ≜ & \text{diag}\left\{-r_{1}{\hat{\lambda }}_{1},-r_{2}{\hat{\lambda }}_{2},-r_{3}{\hat{\lambda }}_{3},-r_{4}{\hat{\lambda }}_{4}\right\}\\ b_{2} & ≜ & {\left[I_{\omega }{\dot{\hat{\omega }}}_{1}-T_{1},I_{\omega }{\dot{\hat{\omega }}}_{2}-T_{2},I_{\omega }{\dot{\hat{\omega }}}_{3}-T_{3},I_{\omega }{\dot{\hat{\omega }}}_{4}-T_{4}\right]}^{T}\\ {\text{Q}}_{2} & ≜ & \text{diag}\left\{q_{21},q_{22},q_{23},q_{24}\right\}\\ {\text{W}}_{2} & ≜ & \text{diag}\left\{w_{21},w_{22},w_{23},w_{24}\right\}. \end{array}$$

Noted that the matrix **A**_*rls*,2_ is singular when one of the slip ratios is zero. It can be understood that the tracking stiffness cannot be identified when there is no traction force. Moreover, the angular rates of four tires are directly measured by tachometers (see Table 1) and conditioned by the alpha-beta filter. The slip ratios are calculated using the measurements from the sensor fusion system and the tachometers. The applying wheel torque is assumed to be obtained from torque sensors.

#### 5.2.3. Cornering Stiffness Identification

The longitudinal and lateral dynamics in the vehicle model Equations (13) and (15)–(17) can be rearranged as:

(22) $$Q_{3}A_{\text{rls},3}W_{3}W_{3}^{-1}{\bar{x}}_{3}=Q_{3}b_{3}$$

where

$$\begin{array}{lll} {\bar{x}}_{3} & ≜ & {\left[{\bar{C}}_{\alpha ,f},{\bar{C}}_{\alpha ,r}\right]}^{\text{T}}\\ {\text{A}}_{\text{rls},3} & ≜ & \left[\begin{array}{cc} -\frac{{\hat{\alpha }}_{1}+{\hat{\alpha }}_{2}}{2}\sin\frac{\delta_{1}+\delta_{2}}{2} & 0\\ \frac{{\hat{\alpha }}_{1}+{\hat{\alpha }}_{2}}{2}\cos\frac{\delta_{1}+\delta_{2}}{2} & \frac{{\hat{\alpha }}_{3}+{\hat{\alpha }}_{4}}{2} \end{array}\right]\\ b_{3} & ≜ & \left[\begin{array}{c} {\bar{m}}_{\text{tot}}\left({\ddot{\hat{x}}}_{a}-\dot{\hat{y}}{\dot{\hat{\psi }}}_{v}-{\hat{G}}_{x}\right)-\sum {\bar{C}}_{\lambda ,1}{\bar{\lambda }}_{i}\cos\delta_{i}\\ {\bar{m}}_{\text{tot}}\left({\ddot{\hat{y}}}_{a}-\dot{\hat{x}}{\dot{\hat{\psi }}}_{v}-{\hat{G}}_{y}\right)-\sum {\bar{C}}_{\lambda ,1}{\bar{\lambda }}_{i}\sin\delta_{i} \end{array}\right]\\ {\text{Q}}_{3} & ≜ & \text{diag}\left\{q_{31},q_{32}\right\}\\ {\text{W}}_{3} & ≜ & \text{diag}\left\{w_{31},w_{32}\right\}. \end{array}$$

Noted that the matrix **A**_*rls*,3_ is singular when the summation of the front (or rear) two slip angles is zero or the summation of the front steering wheel angles is zero. Again, it can be understood that the cornering stiffness cannot be identified when there is no lateral force. Moreover, the slip angles are calculated using the measurements from the sensor fusion system and the steering wheel angle.

#### 5.2.4. Moment of Inertia Identification

The rotational dynamics in the vehicle model in Equation (13) can be rearranged as:

(23) $${\text{Q}}_{4}{\text{A}}_{\text{rls},4}{\text{W}}_{4}{\text{W}}_{4}^{-1}{\bar{x}}_{4}={\text{Q}}_{4}b_{4}$$

where

$$\begin{array}{ccc} {\bar{x}}_{4} & ≜ & {\left[{\bar{I}}_{x},{\bar{I}}_{y},{\bar{I}}_{z}\right]}^{\text{T}}\\ {\text{A}}_{\text{rls},4} & ≜ & \left[\begin{array}{ccc} {\dot{\hat{\omega }}}_{x} & -{\hat{\omega }}_{y}{\hat{\omega }}_{z} & {\hat{\omega }}_{y}{\hat{\omega }}_{z}\\ {\hat{\omega }}_{z}{\hat{\omega }}_{x} & {\dot{\hat{\omega }}}_{y} & -{\hat{\omega }}_{z}{\hat{\omega }}_{x}\\ -{\hat{\omega }}_{x}{\hat{\omega }}_{y} & {\hat{\omega }}_{x}{\hat{\omega }}_{y} & {\dot{\hat{\omega }}}_{z} \end{array}\right]\\ b_{4} & ≜ & {\left[M_{x},M_{y},M_{x}\right]}^{\text{T}}\\ {\text{Q}}_{4} & ≜ & \text{diag}\left\{q_{41},q_{42},q_{43}\right\}\\ {\text{W}}_{4} & ≜ & \text{diag}\left(w_{41},w_{42,}w_{43}\right) \end{array}$$

In Equation (23), the angular velocities and accelerations are provided by the alpha-beta filter via the input from the gyroscope measurements.

## 6. Vehicle Dynamics Prediction

**Figure 2.**
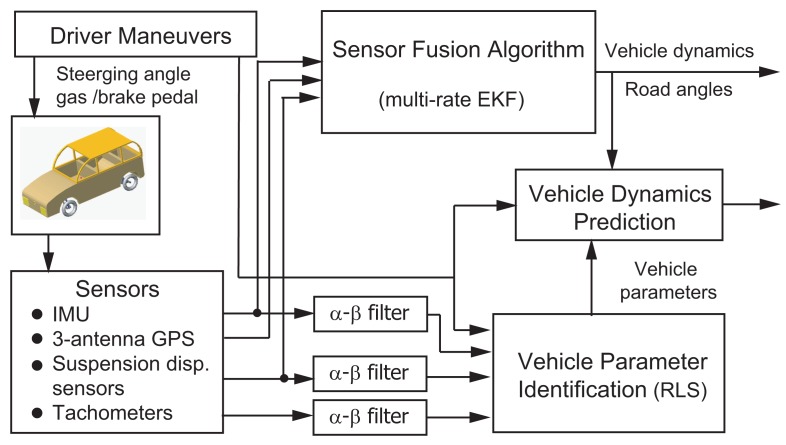
Block diagram of the vehicle dynamics prediction system.

From a system observability viewpoint [30], if both the governing equations of a dynamic system and state values at a time instant are given, the state values at any time instant can be calculated accordingly. Stemming from this concept, one can predict the vehicle dynamics using a vehicle model and current state values. In this case, the current vehicle dynamics is obtained from the sensor fusion system shown in Equations (7)–(9); the vehicle model for propagating the current vehicle states is shown in Equations (13)–(16); the parameters in that vehicle model is estimated using four RLS algorithms in Equations (20)–(23). The block diagram of this signal processing is shown in Figure 2.

## 7. Simulation Results

**Figure 3.**
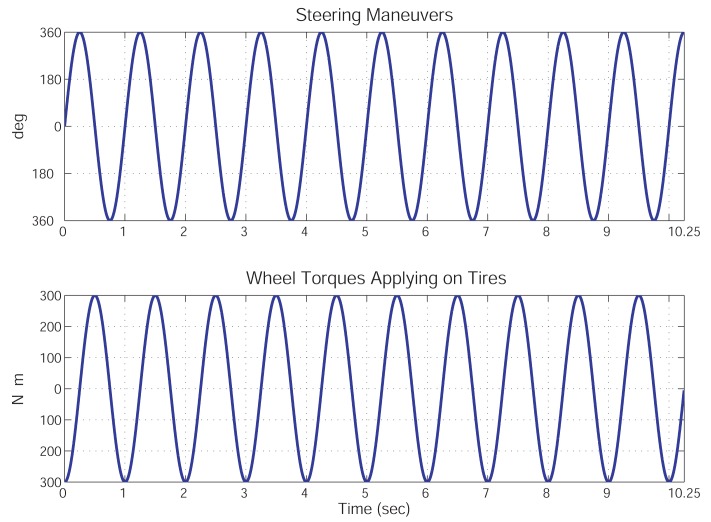
The driving maneuvers for the illustrative simulation. The upper plot is the steering wheel angle and the lower plot is the wheel torques applying on four tires. The frequency is 1 Hz.

Numerical simulations are used to demonstrate the feasibility of the proposed dynamics prediction method. In these simulations, a vehicle moves at a longitudinal speed of 90 km/h. The steering wheel angles and the generated tire torques are both varying with time at the frequency of 1 Hz (see, Figure 3). The road bank angle is 2° and the road grade angle is −2°. A full-state vehicle model, which is a nonlinear 6 DOF vehicle model and consists of 20 states and road angles [7], is used to mimic the real vehicle dynamics on this slope road. This full-state vehicle model differs from the vehicle model shown in Equation (13) only in the tire model, wherein the nonlinear tire model “magic formula” [26] is used. The selected sensors and their output characteristics are listed in Table 1. The sampling frequency of the simulations is 100 Hz. No other disturbance is applied to the vehicle system unless otherwise specified.

### 7.1. Vehicle Dynamics Estimations

**Figure 4.**
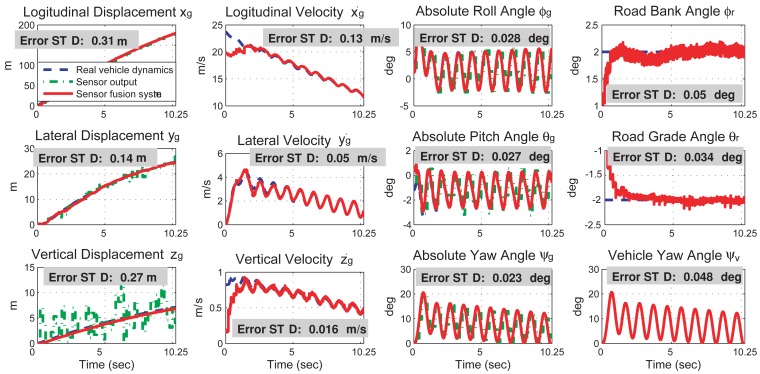
Comparisons of the vehicle dynamics from the simulated vehicle dynamics, the sensor outputs, and the sensor fusion system outputs. The vehicle dynamics are presented in the global frame. The error standard deviations are calculated from the 5th second to the 10th second.

The simulation results of the proposed sensor fusion system are shown in Figure 4, where the state values from the full-state vehicle model are drawn in dashed-blue line, the sensor outputs are drawn in dashed-dotted-green line, and the state values from the output of the sensor fusion system are drawn in solid-red line. Simulation results indicate that the proposed sensor fusion system can accurately obtain the 6 DOF vehicle dynamics and two road angles. The estimation error of each vehicle state, which is defined as the standard deviation of the difference between the simulated vehicle dynamics and the sensor fusion outputs, is also shown in Figure 4.

### 7.2. Vehicle Parameter Identifications

**Figure 5.**
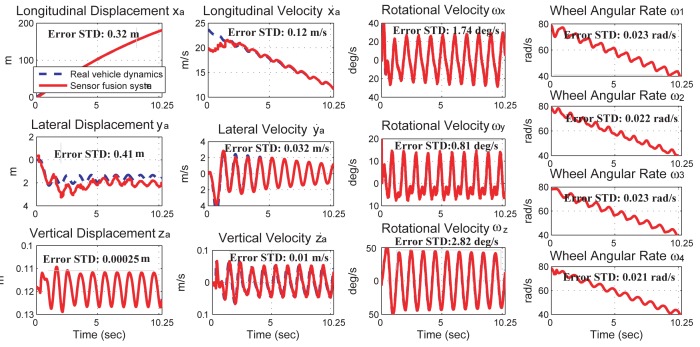
Comparisons of the vehicle dynamics from the simulated vehicle dynamics and the sensor fusion system outputs. The vehicle dynamics are presented in the aux-frame.

**Figure 6.**
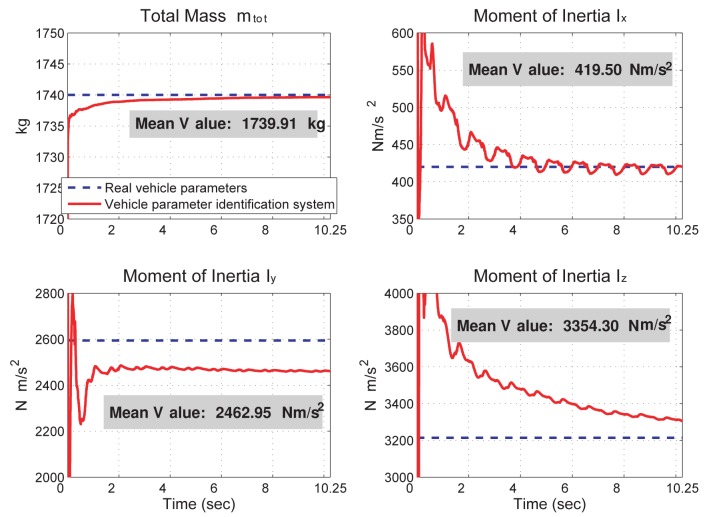
The identification of the vehicle mass and moment of inertia. The mean values are calculated from the 15th second to the 10th second.

**Figure 7.**
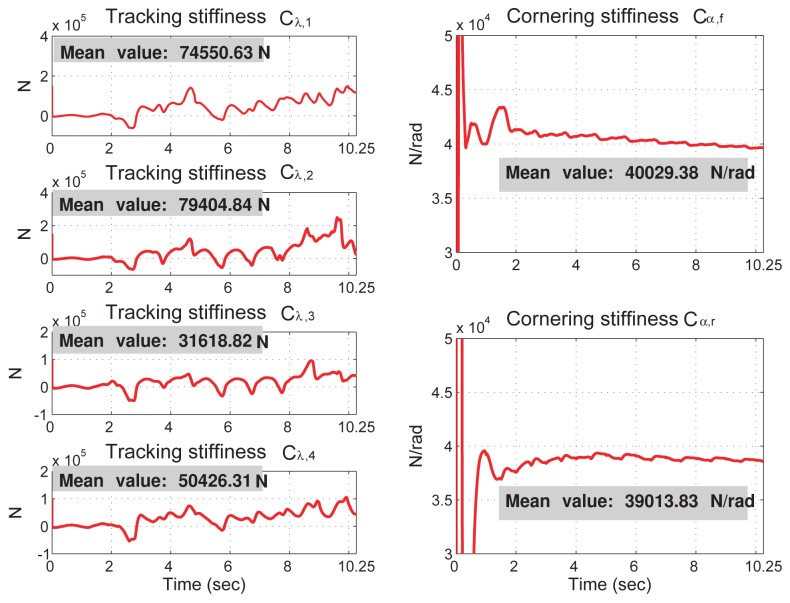
The identification of the tire tracking stiffness and cornering stiffness. The mean values are calculated from the 15th second to the 10th second.

The vehicle dynamics and sensor fusion outputs presented in the global frame (show in Figure 4) are converted into the aux-frame and shown in Figure 5. These state values, along with direct sensor measurements conditioned by the alpha-beta filter, are used for the vehicle parameter identification. The identification results are shown in Figures 6 and 7, where the identified vehicle parameters are drawn in the solid-red line, and the real vehicle parameters are drawn in the dashed-blue line. The “relative inaccuracy” of estimation, which is defined as (real value — identified value)/(real value) [31], of the mass and moment of inertial are calculated to be (*m̅_tot_, I̅_x_, I̅_y_, I̅_z_*) = (5.17 × 10^−3^%, 0.12%, 5.05%, 4.37%). The estimation accuracy is good mainly because the incorporated suspension displacement sensors are relatively accurate. On the other hand, as shown in Figure 7, the estimation of tracking stiffness and cornering stiffness do not converge well and because there is no corresponding tracking stiffness and cornering stiffness in the full-state vehicle model. The feasibility of the tire stiffness estimation is discussed in the next section.

### 7.3. Vehicle Dynamic Predictions

**Figure 8.**
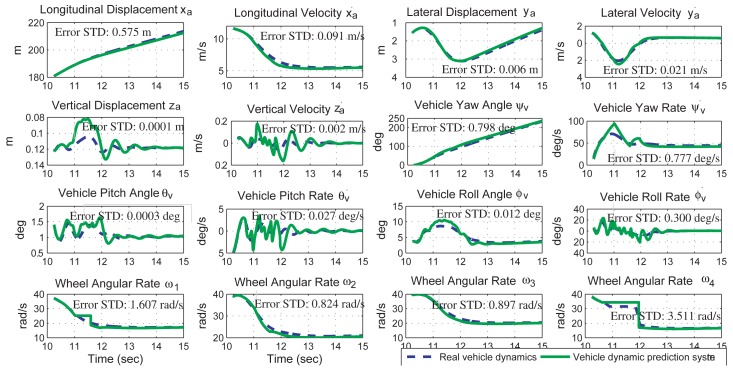
Predictions of the vehicle dynamics in a left-hand turn event. The prediction inaccuracy is 0.51% on average, calculated from the 10.25th second to the 15th second.

Continuing from previous simulations, the driver is assumed to hold still the steering wheel and the gas/brake pedal at the time instant 10.25 s to do a left-hand turn on the road. The prediction system is turned on at the 10.25 s to predict the vehicle dynamics for the next 4.75 s, using the vehicle dynamics from the sensor fusion system at the 10.25 s and the vehicle model with the parameters identified from Figures 6 and 7. Since the steering wheel and the gas/brake pedal are the same during 10.25 to 15 s, both the vehicle dynamics and the prediction results during this period can be shown in the same plot for comparison. In Figure 8, the real vehicle dynamics are drawn in the dashed-blue line, and the predicted vehicle dynamics are drawn in the solid-green line. According to the simulation results, the proposed method can predict the vehicle dynamics accurately. The prediction error of each state can be found in the plot. The relative-inaccuracy of this prediction, averaged from vehicle displacements (*x_a_, y_a_, z_a_*) and vehicle attitude (*ψ_v_, θ_v_, ϕ_v_*), is 0.51%.

**Figure 9.**
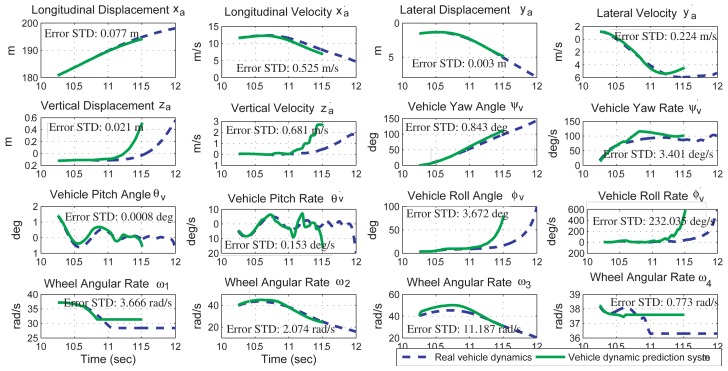
Predictions of the vehicle dynamics in a rollover event. The prediction system successfully predicts the rollover incident. The prediction inaccuracy is 27.3% on average, calculated from the 10.25th second to the 11.5th second.

In another example, the driver holds the steering wheel still but generate 1,000 *N* · *m* torques on two tires on the right hand side (*T*_2_ = *T*_3_ = 1,000 *N* · *m*). In that case, the vehicle is likely to rollover due to the excess yaw moment applying to the vehicle. The simulation results (see Figure 9) show that the vehicle roll angle is larger than 90° at the 11.5 s, which indicates a rollover incident. The dynamic prediction system can predict the rollover event. The relative-inaccuracy of this prediction is 27.3%, which is calculated from 10.25 s to 11.5 s and averaged from vehicle states including vehicle displacements (*x_a_, y_a_, z_a_*) and vehicle attitude (*ψ_v_, θ_v_, ϕ_v_*). The prediction accuracy is worse than that of the previous case. The reason is discussed in the following section.

## 8. Discussion

**Figure 10.**
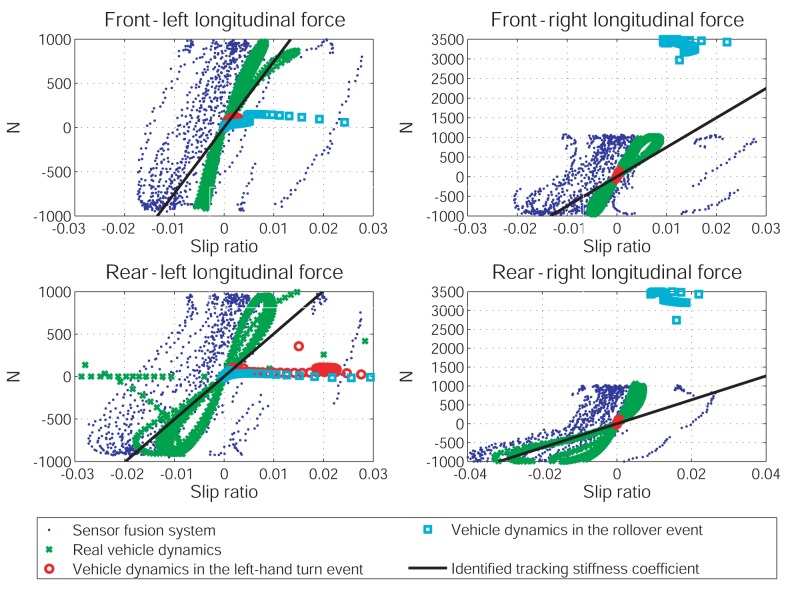
The relations between the slip ratio and the longitudinal tire adhesive force.

**Figure 11.**
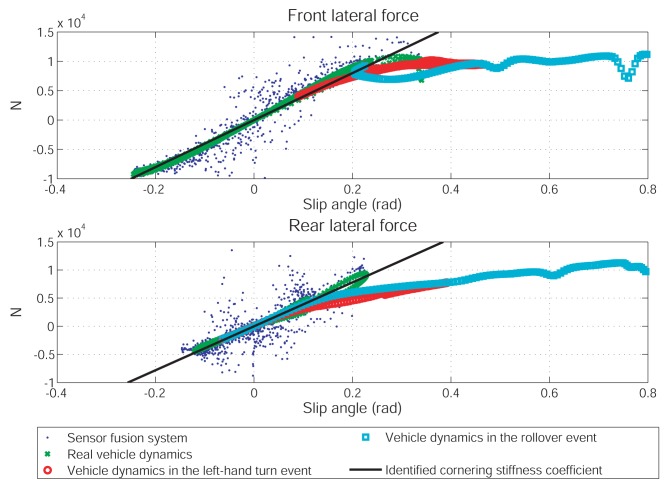
The relations between the slip angle and the lateral tire adhesive force.

Although the vehicle rollover accident can be foreseen in this case, the prediction is a bit inaccurate. According to the parameter identification results shown in Figures 6 and 7, this error is likely due to the model difference used in the prediction process and in the simulated vehicle dynamics. In turns, it leads to two possibilities: (1) the vehicle dynamics estimated from the sensor fusion system are not accurate enough for the traction/cornering stiffness identification; (2) the adhesive tire forces in the rollover event are located in the nonlinear regime and the linear tire model is inadequate to describe them. To clarify this, Figure 10 presents the relation between longitudinal tire force and slip ratio, and Figure 11 presents the relation between lateral tire force and slip angle. As shown in Figure 10, the longitudinal tire force estimated from the sensor fusion system (drawn in blue dots) and from the simulated vehicle dynamics (drawn in green stars) are not the same. Thus, the sensor noise associated with the selected sensors does affect the estimation of longitudinal dynamics to certain extent. In turns, the identified traction stiffness (drawn in solid-black line) cannot be accurate. On the other hand, as shown in Figure 11, the lateral tire force estimated from the sensor fusion system are close to the simulated vehicle dynamics. Thus, the identified cornering stiffness is more accurate than the identified traction stiffness.

The tire forces of two prediction cases are also shown in Figures 10 and 11. The tire forces are drawn in red circles for the left-turn event and in cyan squares for the rollover event. The tire forces of the left-hand turn event are very close to the forces calculated from the identified traction/cornering stiffness, except for the force at the rear-left tire. Thus, the prediction of the left-hand turn dynamics is quite accurate. On the other hand, the tire forces of the rollover event are far from the forces calculated from the identified traction/cornering stiffness. Thus, the prediction of the rollover dynamics is a bit inaccurate.

From above discussion, one may propose using nonlinear tire models, such as Pacejka's magic formula [26] or Dugoff tire model [27], for the friction coefficient identifications and dynamics predictions. Our experiences show that it is possible to identify more parameters of a nonlinear tire model as long as the nonlinear tire adhesive forces are present in the measured vehicle dynamics (persistent excitation condition). However, the tire adhesive forces are within in the linear regime in most driving conditions. In that case, including a nonlinear tire model in the identification process would not gain any advantage but cause convergence problems.

This vehicle parameter identification is challenging mainly because the system has a low degree-of-observability [32,33], and it gets worse when large amount of noise is present in the sensor measurements. This effect can be investigated by the signal-to-noise ratio (SNR), where the signal is referred to as the estimated vehicle dynamics from the sensor fusion system, while the noise is referred to as the estimation error. To show how the SNR affects this parameter identification, we use the identification of the moment of inertia as an example. As shown in Table 2, the estimation error can be minimized when the SNR is large, and the relative error approaches (1.94%, 2.68%, 0.33%) for (*I̅_x_, I̅_y_, I̅_z_*). The best estimation accuracy is limited by the numerical errors and the model errors from the linear tire model assumption. To enlarge the SNR, either the range of vehicle dynamics needs to be enlarged or the noise from the sensor measurement needs to be minimized. The vehicle dynamics cannot have a large span due to its strong stability. On the other hand, using high-end sensors would reduce the noise but incur higher cost.

One alternative to improve this parameter identification is to increase the degree-of-observability by choosing proper weighting and scaling matrices shown in Equation (19). To show the effectiveness of this approach, we use the identification of the moment of inertia as an example and assume no noise in the sensor measurement. The choice of weighting matrix (**Q**_4_) changes the minimum eigenvalue of the estimation matrix $\left({\text{A}}_{\text{rls},4}^{\text{T}}{\text{Q}}_{4}^{\text{T}}{\text{Q}}_{4}{\text{A}}_{\text{rls},4}\right)$ and results in different degree-of-observability [32,33]. Table 3 shows the larger minimum eigenvalues of the resulting matrix, the better parameter observability and the faster convergence rate of the parameter identification.

## 9. Conclusions

A vehicle dynamics prediction system, consisting of a kinematics-based sensor fusion system and a vehicle parameter identification system, is proposed and verified by simulation results. The sensor fusion system can obtain the 6 DOF vehicle dynamics and the two road angles accurately. The estimation error for each vehicle dynamics is shown in Figure 4. The vehicle parameter identification system uses the dynamics information from the sensor fusion system to identify ten vehicle parameters in real time. The identification inaccuracy of the vehicle mass and moment of inertia is less than 5.05%. Using the vehicle dynamics from the sensor fusion system and the vehicle model from the parameter identification system, the prediction system successful predicts the vehicle dynamics in a left-hand turn event and a rollover event. The prediction inaccuracy is 0.51% in the left-hand turn event and 27.3% in the rollover event.

The prediction accuracy of the rollover event is worse than that of the left-hand turn event. It is mainly because the identified linear tire model cannot accurately describe the nonlinear tire adhesive force in the rollover event. Using a nonlinear tire model for the dynamics prediction is possible but not practical in this case, because the nonlinear tire behaviors are not excited in normal vehicle maneuvers.

The prediction accuracy of two scenarios suggests that modeling error of the unsprung mass system may greatly affect the accuracy of the parameter identification and thus the dynamics predictions. Therefore, a detail modeling and/or real-time system identification of the unsprung mass system may be needed to improve the feasibility of this approach. Besides, this research also shows that the vehicle parameter identification is challenging because the system has a low degree-of-observability. Therefore, increasing the SNR of the sensor systems and careful designs of the weighting matrix of the identification algorithm are recommended.

## Figures and Tables

**Table 1.** Sensor output rates and noise characteristics.

	Output frequency	Noise

		Standard deviation
GPS	5 Hz	0.4°
(attitude measurement)		
GPS	5 Hz	horizontal: 1 m
(position measurement)		vertical: 3 m
Suspension	1 Hz	0.001 m
displacement sensor		
Accelerometer	1 kHz	0.02 m/s^2^
Gyroscope	1 kHz	0.08°/s
Tachometer	1 kHz	2°/s

The measurement bias is not considered.

**Table 2.** The relations between the estimation error of sensor fusion system and the relative inaccuracy of the parameter identification.

relative inaccuracy		*I̅_x_*	*I̅_y_*	*I̅_z_*
	infinite	1.94%	2.68%	0.33%
SNR	30 dB	2.50%	14.62%	1.52%
	20 dB	14.91%	60.28%	10.41%
	10 dB	69.05%	93.63%	54.11%

**Table 3.** Different weighting matrices result in different convergence rate.

	convergence rate a	*I̅_x_*	*I̅_y_*	*I̅_z_*
	diag{0.625, 0.625, 0.625}	0.08 s	1.63 s	0.24 s
Q_4_^b^	diag{0.125, 0.125, 0.125}	6.19 s	18.2 s	3.32 s
	diag{0.025, 0.025, 0.025}	36 s	Nan c	25 s
	diag{0.005, 0.005, 0.005}	Nan c	Nan c	Nan c

a The convergent rate is defined at the time when estimated value reaches 90% of the real value.

b **Q**_4_ and **W**_4_ are both designed as a diagonal matrix. **Q**_4_ varies in each case, while **W**_4_ is kept the same as diag{1, 1, 1}.

c The value “Nan” means that the convergence time is too long to calculate.
